# Diagnostic accuracy of C-reactive protein and procalcitonin in the early detection of infection after elective colorectal surgery – a pilot study

**DOI:** 10.1186/1471-2334-14-444

**Published:** 2014-08-16

**Authors:** Joana Silvestre, Jorge Rebanda, Carlos Lourenço, Pedro Póvoa

**Affiliations:** Polyvalent Intensive Care Unit, São Francisco Xavier Hospital, CHLO, Lisbon, Portugal; CEDOC, Faculty of Medical Sciences, New University of Lisbon, Lisbon, Portugal; Department of Surgery I, São Francisco Xavier Hospital, CHLO, Lisbon, Portugal

**Keywords:** C-reactive protein, Procalcitonin, Biomarkers, Colorectal surgery, Surgical infections

## Abstract

**Background:**

Colorectal surgery is associated with postoperative infectious complications in up to 40% of cases, but the diagnosis of these complications is frequently misleading, delaying its resolution. Several biomarkers have been shown to be useful in infection diagnosis.

**Methods:**

We conducted a single-centre, prospective, observational study segregating patients submitted to elective colorectal surgery with primary anastomosis, CRP and PCT were measured daily. We compared infected and non-infected patients.

**Results:**

From October 2009 to June 2011, a total of 50 patients were included. Twenty-one patients developed infection. PCT and CRP before surgery were equally low in patients with or without postoperative infectious complications. After surgery, both PCT and CRP increased markedly. CRP time-course from the day of surgery onwards was significantly different in infected and non-infected patients (P = 0.001) whereas, PCT time-course was almost parallel in both groups (P = 0.866). Multiple comparisons between infected and non-infected patients from 5^th^ to 9^th^ postoperative days (POD) were performed and CRP concentration was significantly different (P < 0.01, Bonferroni correction), on the 6^th^, 7^th^ and 8^th^ POD. A CRP concentration > 5.0 mg/dl at the D6 was predictive of infection with a sensitivity of 85% and a specificity of 62% (positive likelihood ratio 2.2, negative likelihood ratio 0.2).

**Conclusions:**

After a major elective surgical insult both CRP and PCT serum levels increased independently of the presence of infection. Besides serum CRP time-course showed to be useful in the early detection of an infectious complication whereas PCT was unhelpful.

**Electronic supplementary material:**

The online version of this article (doi:10.1186/1471-2334-14-444) contains supplementary material, which is available to authorized users.

## Background

Elective colorectal surgery is associated with postoperative infectious complications in up to 40% of the cases [1, 2]. Despite recent advances in both surgical technique and perioperative care, infectious complications remain a major clinical problem in colorectal surgery, contributing to significant postoperative morbidity, increased mortality, prolonged hospital stay and additional costs [3–6]. As a result, early diagnosis of the infectious complications is a crucial step in order to initiate treatment as soon as possible [7].

Nonetheless, the diagnosis of infectious complications after elective colorectal surgery is frequently misleading, delaying its resolution. Consequently the availability of an early sensitive and specific marker of postoperative infectious complications would be of great interest [8].

Several biomarkers of infection, namely C-reactive protein (CRP) and procalcitonin (PCT), have been shown to be useful in the diagnosis of infection in different clinical settings as well as in the assessment of its response to antibiotic therapy [9–11].

C-reactive protein has been studied by several authors as an early predictor of abdominal septic complications after esophageal, pancreatic and rectal resection with sensitivities and specificities between 65 and 80% [12–15]. Reith et al. used PCT to identify patients with postoperative complications after elective surgery of the colon and the aorta [16]. In this study, on the first postoperative day (POD) serum PCT was higher in patients with complications (6.9 versus 1.12 ng/mL) [16]. However, the preoperatively PCT levels were already higher in the group with complication.

C-reactive protein (CRP) is one of these biomarkers and probably the most widely used. In different infections and clinical settings, the course of relative CRP variations and the CRP ratio, can discriminates, early in the clinical course, survivors from non-survivors [17, 18][19]. The identification of the individual pattern of CRP ratio response to antibiotic therapy appears to be a reflection of the clinical course of infection independently of other possible confounders [20].

Recently Oberhofer and colleagues [21] demonstrated in a prospective study in patients submitted to elective colorectal surgery that CRP concentrations were not inferior to PCT levels in identifying patients with infectious postoperative complications. In this study ROC curve analysis showed that CRP concentrations on POD 3 and PCT concentrations on POD 2 had similar predictive values for the development of infectious complications (AUC 0.746 and 0.750, respectively).

To the best of our knowledge, this is the only study that compared the diagnostic accuracy of CRP and PCT for early detection of postoperative complications in patients undergoing colorectal surgery.

The present study was designed to assess the value of serum CRP and PCT time course in the postoperative setting of elective colorectal surgery with primary anastomosis and its potential in detecting infectious postoperative complications.

## Methods

The present study was a prospective, single centre, observational study conducted during a 21 months period, between October 2009 and June 2011, in the General Surgery Department of São Francisco Xavier Hospital. Our hospital is a central and university hospital of Lisbon that belongs to a Hospital of 900 beds. Fifty patients with elective colon resections with primary anastomosis were prospectively included in the present study. Inclusion criteria were age >18 and elective colon resections. Patients were excluded if they were on systemic antibiotics at the time of surgery, if it was not able to obtain an informed consent and if they were in other clinical trial. The West Lisbon Hospital Centre Ethics Committee previously approved the study. Informed consent was obtained from all patients or legal representative before surgery.

### Data collection and definitions

Data on patient demographics, surgical procedures, postoperative mortality and morbidity were prospectively collected from medical records. The baseline characteristics of the patients enrolled in the study are detailed in Table 1.

**Table 1:** **Clinical and demographic characteristics of the patients treated by colorectal resection**

	Non-infected N = 29	Infected N = 21	***P***
Age, yrs	70.3 ± 10.9	70.7 ± 7.2	0.873
Male sex (M/F)	18/11	14/7	0.042
Body mass index	26.5 ± 5.0	28.5 ± 6.3	0.176
Charlson score, points	4.45 ± 1.64	3.86 ± 1.42	0.19
Co morbidities, N (%)	26 (89.7)	19 (90.5)	0.924
Diagnosis			0.971
Cancer, N (%)	24 (82.8)	17 (80.9)	
Diverticular disease, N (%)	4 (13.8)	3 (14.3)	
Other, N (%)	1 (3.4)	1 (4.8)	
Location of the disease			0.173
Ascending colon, N (%)	13 (44.8)	5 (23.8)	
Descending colon, N (%)	3 (10.3)	1 (4.8)	
Sigmoid / Rectum, N (%)	13 (44.8)	15 (71.4)	
Bowel preparation, N (%)	19 (65.5)	18 (85.7)	0.191
Antibiotic prophylaxis, N (%)	25 (86.2)	20 (95.2)	0.383
Surgical intervention			0.2
Right hemicolectomy, N (%)	13 (44.8)	4 (19.1)	
Left hemicolectomy, N (%)	3 (10.3)	2 (9.5)	
Sigmoidectomy, N (%)	6 (20.6)	9 (42.9)	
Total colectomy, N (%)	0	1 (4.8)	
Anterior resection, N (%)	7 (24.1)	4 (19.4)	
Other, N (%)	0	1 (4.8)	
Type of infection, N			
Anastomotic leak		1	
Intraabdominal abcess		1	
Surgical site infection		16	
Central line infection		1	
Pneumonia		1	
Urinary tract infection		1	
Infection diagnosis, day		7.2 ± 2.3	
Admission in ICU, N (%)	7 (50)	7 (50)	0.534
Length of stay, days	11 [7]	21 [14]	0.001
Mortality, N (%)	0	2 (9.5)	0.171
Preoperative CRP, mg/dL	0.39 [0.48]	0.5 [2.19]	0.473
Preoperative PCT, ng/dL	0.08 [0.04]	0.08 [0.07]	0.471
Preoperative white cell count, x10^6^/L	6900 ± 1300	7000 ± 1900	0.891
Preoperative platelets, x10^9^/L	242 ± 71	259 ± 70	0.389
Preoperative body temperature, °C	36.2 ± 0.4	36.4 ± 0.5	0.221

ICU – Intensive Care Unit; CRP – C-reactive Protein; PCT – Procalcitonin. Data presented as mean ± SD or median [IQR].

Postoperative infectious complications were defined as follows: anastomotic leakage (AL), abscess, surgical site infection, pneumonia, urinary tract infections and central line infections.

Patients underwent further postoperative diagnostic test or treatment only in case of symptoms or signs of an infectious complication. An AL was verified either by radiography enema performed with computed tomography scan, x-ray or by endoscopy. Abscesses were verified by purulent drainage or during re-laparotomy. Surgical site infection was diagnosed by the presence of clear signs of inflammation at the wound margin or purulent drainage from the wound [22]. Diagnosis of central line infection required positive blood cultures and cultures from the catheter tip with the same microorganism [23]. Pneumonia was diagnosed by the presence of new pulmonary infiltration chest radiography or in chest CT scan accompanied by clinical symptoms of the lower respiratory tract or on physical or laboratory exam [24]. Urinary tract infection was defined by positive urine sediment analysis combined with fever and/or leukocytosis [25].

Pneumonia and UTI were treated with antibiotics; central line infections were treated with antibiotics and removal of the catheter; surgical site infections were treated with drainage and antibiotics; AL and abdominal abscess were treated with re-laparotomy and antibiotics.

Serum CRP, PCT, white cell count (WCC), platelets and higher body temperature were recorded routinely before surgery, and on POD 1 to 12 or before if the patient was discharge earlier.

### Biomarkers measurements

Measurement of CRP was performed by an immunoturbidimetric method (Tina-quant CRP; Roche Diagnostics, Mannheim, Germany). The precision of the assay measured by the intra- and inter-assay coefficient of variation was < 7%, the sensitivity of the method was 0.1 mg/dL and the detection limit was 0.3 mg/dL.

Procalcitonin was quantified by sensitive time-resolved amplified cryptate emission assay (Kryptor PCT, B.R.A.H.M.S AG, Hennigsdorf, Germany). The precision of the assay measured by the intra- and inter-assay coefficient of variation was between 2 and 3%, the sensitivity of the method was 0.01 ng.mL^−1^ and the detection limit was 0.06 ng.mL^−1^.

Comparison between infected and non-infected patients after elective colorectal surgery with primary anastomosis was performed.

### Statistics

Results are expressed as the mean ± standard deviation unless stated otherwise. To assess differences between the two main groups, infected and non-infected patients, the Student's t test and the Mann–Whitney U test were used for continuous variables and the χ2 test was used for categorical variables. Time dependent analysis of different variables was performed with a general linear model, univariate, repeated-measures analysis using a split-plot design approach.

Median values with interquartile range were used for graphical visualization. Logistic regression models were used to determine if each biomarker from the POD5 to POD9 was associated with complications. Bonferroni correction was used to counteract the problem of multiple comparisons [26].

The diagnostic accuracy was evaluated with area under the curve (AUC), using the ROC methodology [27]. The AUCs were computed using the non parametric trapezoidal method and their 95% confidence limits were computed according to method established by DeLong [28].

Results were reported as the odds ratio with a 95% confidence interval. Significance was accepted for P < 0.05 unless otherwise stated. Data were analyzed using PASW Statistics v.18.0 (SPSS, Chicago, IL).

## Results

### Baseline and outcomes

From October 2009 to June 2011, a total of 50 patients were prospectively included.

Infectious complications were diagnosed in 21 patients (42%): sixteen surgical site infections, one AL, one intra-abdominal abscess and three extra-abdominal infections. The median day of diagnosis of complications was POD7 (interquartile range (IQR) 5–12). Infection was less frequent in men (28% vs. 72%, P = 0.042).

Diagnosis, comorbidities and surgical procedures were similar in patients with and without infectious complications.

Of the 50 patients, 14 (28%) were admitted in the Intensive Care Unit and two (4%) patients died due to nosocomial infections. Clinical and demographics characteristics are expressed in Table 1.

### White cell count, platelets and body temperature time course

**Figure 1:**
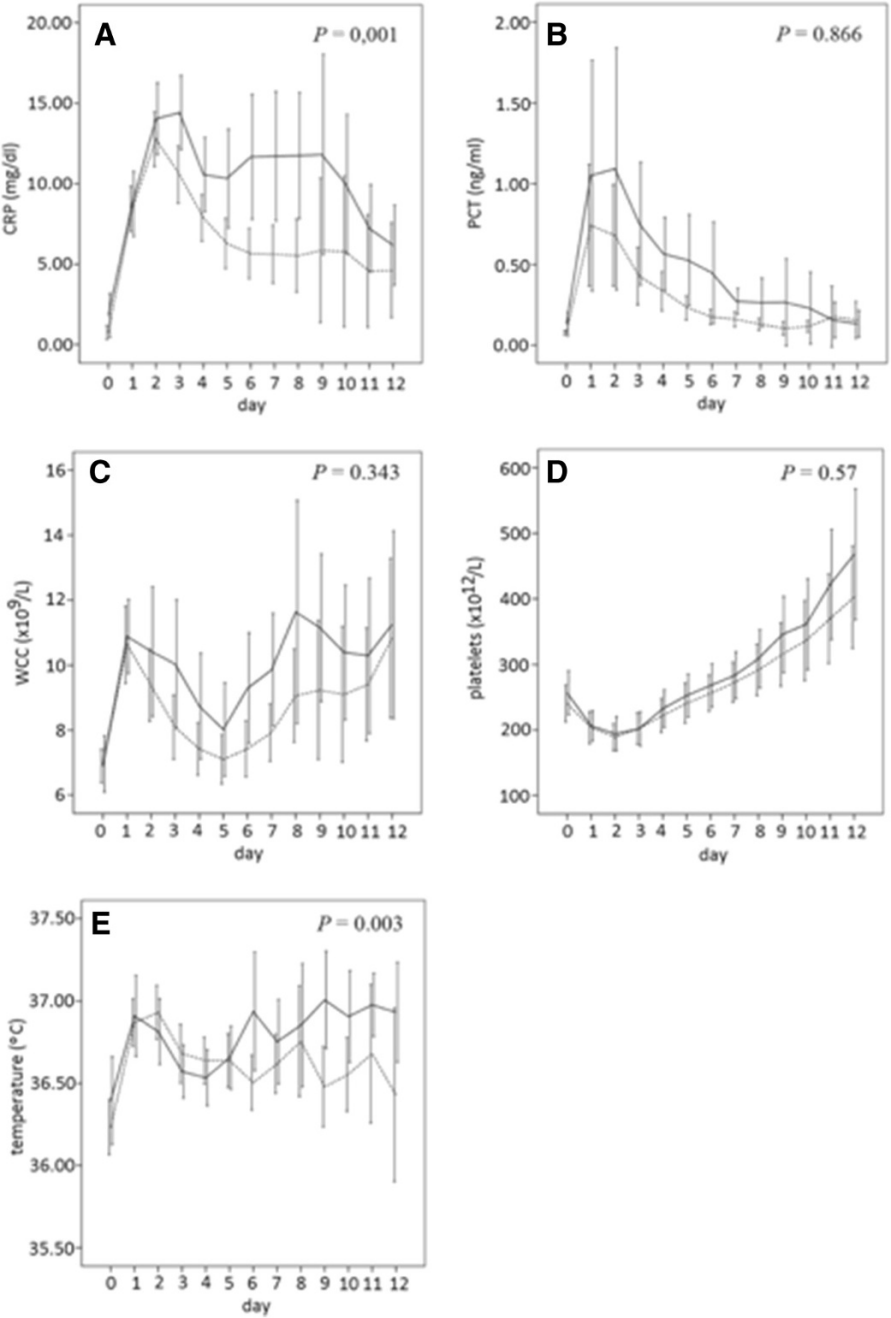
**Observed means of C-reactive protein (A), procalcitonin (B), white cell count (C), platelets (D) and temperature (E) during the first 12 days after elective colo-rectal surgery for non-infected (dashed line) and infected (solid line) patients.** Error bars represent point-wise 95% confidence intervals.

Mean preoperative values of WCC, platelets and body temperature were similar in infected and non-infected patients (Table 1). In the POD1, WCC increased in both groups, being slightly higher in the infected group although not reaching statistical significance (Figure 1C).Platelets decreased after surgery, reaching the lower value on the POD2 and gradually increased thereafter in both groups (Figure 1D).Body temperature was significantly higher on the POD6 in patients who developed a post-operative infection complication (P = 0.003) (Figure 1E), however the absolute difference between the infected and non-infected group was only 0.3°C. These findings promote the use of biomarkers to help the discrimination between infection and non-infection promoting an earlier focus control, since clinical data are many times insufficiently.

### CRP and PCT time course

The levels of CRP and PCT before surgery were very low and similar in patients with an uneventful course as well as in those that went on to develop an infectious complication (0.1 ± 0.06 vs. 0.07 ± 0.04 ng/ml; 1.81 ± 2.83 vs. 0.72 ± 1.12 mg/dl, respectively). After surgery, in the POD1, both CRP and PCT increased : CRP increased more than 15× and peaked at 48 hrs; PCT increased around 10× the basal level and peaked at 24 to 48 hrs (Figure 1A and B).The CRP time-course from the day of surgery onwards was significantly different in infected and non-infected patients (P = 0.001). After the postoperative peak at 48 hrs, CRP decreased persistently in patients with an uneventful postoperative course (Figure 1A). In opposition, in those that will develop an infectious complications CRP decreased from during the POD2 and POD3 until reaching a concentration plateau between POD4 to POD9. After resolution of infection complication, CRP decreased again reaching at POD12 values close to those of non-infected patients. The lack of decrease in CRP levels in the post operative period is a predictor of post-operative infections in patients submitted to colorectal surgery. In opposition, the PCT time-course was almost parallel in both groups, infected and non-infected (P = 0.866) (Figure 1B).

To assess the diagnostic performance of each biomarker, we performed multiple comparisons between infected and non-infected patients from the POD5 to POD9. The CRP concentration was significantly different (P < 0.01, after Bonferroni correction), on the POD6, POD7 and POD8. The CRP levels predicted the infection in general one-day earlier, since the mean of postoperative infection occurred on POD7.

The results of the CRP ROC analysis to assess the diagnostic performance of surgical infectious complications are shown on Table 2. As early as the POD6, a CRP concentration >5.0 mg/dl was associated to with occurrence of infectious complications, with a sensitivity of 85% and a specificity of 62% (positive likelihood ratio 2.2, negative likelihood ratio 0.2).

**Table 2:** **Receiver operating characteristics curve analysis of C-reactive protein during the postoperative course of patients after colorectal surgery**

	AUC (95% CI)
CRP POD 6	0.740 (0.599-0.880)
CRP POD 7	0.730 (0.583-0.878)
CRP POD 8	0.750 (0.591-0.909)

CRP - C-reactive Protein; POD – Postoperative day; AUC – area under curve; CI – confidence intervals.

## Discussion

The early identification of patients who developed infectious complications is crucial for timely and adequate treatment. Severe sepsis is still a major cause of postoperative morbidity and mortality after major surgery, with an incidence ranging between 9 to 12% and high mortality (42% to 80%) [1, 29–32].

In our study the rate of infectious complications after elective colonic surgery was high, 42%, however the majority (36%) were surgical site infections. An AL was diagnosed in only one patient. The mortality was 4%, similar to the outcomes described in other studies [33, 34].

Septic complications after colorectal resection consist mainly of surgical site infections (up to 40%), pulmonary infections (10%) and urinary infections (5%) [2]. Anastomotic leakage and intraabdominal abcess are the most feared complications and are frequently diagnosed late in the postoperative period since the initial clinical manifestations are very subtle. About 30% of patients admitted to the ICU with intraabdominal infection died, with mortality rates even higher when peritonitis arises as a complication of a previous operative procedure [35, 36].

A method for the early identification of patients at risk for pos-operative infection would be of clinical importance, since clinical signs are usually insensitive and do not allow an early diagnosis. Several biochemical tests are used to identify persistent inflammatory activity in postoperative patients, including CRP, PCT and interleukins.

The early identification of patients at risk for post-operative infection would be of clinical importance, since clinical signs are usually insensitive and do not allow an early diagnosis. Several biochemical tests are used to identify persistent inflammatory activity in postoperative patients, including CRP, PCT and interleukins [37–39].

In our study, only CRP demonstrated to be useful in discriminating between infected and non-infected patients. The CRP levels predicted the infection in general on POD 6–7, one-day earlier, than the (median) day of post operative infection diagnosis. PCT and the other inflammatory markers were not useful in discriminating between infected and non-infected patients. Despite body temperature at POD6 was significantly higher in patients who developed a post-operative infection complication the difference between groups was only 0.3°C and no clinical significance was attributable. Recently, in a similar study Oberhofer et al. [21] demonstrated that both CRP and PCT in the early postoperative period, with a significant difference between patients with and without infectious complications.

In our study, PCT failed to discriminate between infected and non-infected patients. Meyer et al. recently reported in literature that in critically ill surgical patients an increase in PCT levels did not help to predict surgical complications [40].

It is well known that after of the presence a major elective surgical insult both CRP and PCT serum levels markedly increased independently of infection. It is demonstrated that by using a model of systemic inflammation after intravenous endotoxin administration, showed that PCT levels in healthy subjects reached a maximum by 24 h and remained above normal for >7 days. In contrast, CRP had normalized in the same subjects by 7 days [41]. Despite this data clinical studies are controversial Lindberg et al. in a series of 47 patients with major abdominal surgery and a uneventful post-operative course, mean CRP increased in the first 48 hrs and reached half its maximum value on the POD5 whereas PCT declined after 24 hrs [42]. On the other hand Meissner et al. [43] described that peak PCT levels are reached within 24 hours postoperatively and return to normal levels within the first week, depending the degree of PCT elevation on the intraoperative course and the type of the surgical procedure.

In our study the peak of PCT was reached between 24 and 48 hrs, after this period PCT could not discriminate between infected and non-infected patients. In our study after the postoperative peak at 48 hrs, CRP decreased persistently in patients with an uneventful postoperative course and at POD11 CRP was <50% the basal level.

CRP kinetics has been recently described by several authors as a predictive of infectious postoperative complications [15, 44]. CRP has been studied in detecting AL after rectal resection. Two recent studies reported that persistently increased CRP values after POD 2–4 were associated to a later diagnosis of an AL [15–45].These authors found that prolonged elevation and/or a absence of decline in CRP levels were associated with more infectious complications and poor outcome [15, 44].

More recently Warschkow et al. [46] demonstrated that CRP values exceeding 123 mg/l on POD 4 where associated a higher risk of infectious complications. These authors found that CRP level above 143 mg/L had a good diagnostic accuracy to detect infectious complications with an AUC ROC 0.76 [46]. We found similar results and CRP time course showed to be useful in the early detection of an infectious complication after elective colorectal surgery. The highest diagnostic accuracy was observed for CRP measured on POD 8, with an AUC of 0.75. However, already by POD6, that is to say 1 day before the median day of infectious diagnosis, the CRP AUC was 0.74.

Some authors advocate the use of PCT as an early biomarker of infection. Takakura et al. recently reported in 18 patients with surgical site infections (SSI) in patients undergoing elective colorectal resection that PCT was significantly higher than CRP levels [47]. Nevertheless higher PCT levels were found on POD1 that could be due to the surgical insult. Reith et al. found, in a prospective study involving 70 patients, 35 with intra-abdominal colorectal elective surgery, that PCT levels were closely related to postoperative complications (severe pneumonia, ischemia, and AL) [16]. PCT was measured preoperatively and postoperatively from day 1 to 5 and on days 7 and 10. These authors could not found any differences between groups in other markers of infection such as WCC, CRP, IL-6 and body temperature. However preoperative CRP concentrations in complicated patients were already significantly higher compared to those who do not develop complications, questioning if there was underlying infection prior to surgery. Novotny et al. more recently found in 104 patients with secondary peritonitis that PCT ratio appears to be helpful in distinguishing between patients with successful eradication of the septic focus and those with a persisting infectious focus [48]. In this study CRP was not evaluated [48]. Recently Lagoutte et al. in a study that included patients undergoing elective colorectal surgery could not found differences between PCT and CRP for the detection of AL with a better AUC ROC for CRP on POD 4 [49]. Despite this data were not confirmed by the Garcia-Granero group that demonstrated a PCT superiority in the AL diagnosis [50], both studies only study the AL complication and did not performed a time dependent analysis study.

Our data found that PCT time course could not differentiate between patients who developed complications with lower AUC ROC than those found in CRP.

Few studies have investigated the use of PCT in the diagnosis of intra-abdominal infections. While PCT showed promise as a marker to exclude perforation and ischaemia in obstructive bowel syndrome, the utility in acute appendicitis and pancreatitis was limited [51–54].

In 1993, Assicot et al. showed in children that PCT levels were high in severe bacterial infections in contrast with those who had absent, localized or viral infections [55]. Some authors advocate that patients who have a localised infection without a general systemic response do not appear to have high levels of serum PCT. An extrapolation as been made to patients who do not develop a marked systemic inflammatory response syndrome such as the elderly or the malnourished patient, although this has not been tested [56].

In the present study, most of the infected patients had surgical site infections that could be considered a localised infection and as a result could justify the lack of sensitivity for PCT.

Similar to previous published studies, measurements of WCC contribute little to the early detection of infectious surgical complications [46].

Our pilot study has some limitations. It is a single-centre study and the number of clinical events (21 patients with infection complications) is small which limits the statistical power of our analysis. However, as far as we are aware, this is the first prospective study in elective colorectal surgery that evaluate two biomarkers simultaneously, CRP and PCT, with measurements before the surgery and a 12 days follow-up.

Recently other biomarkers like lipopolysaccharide-binding protein (LBP) have been study as screening tools for AL and increased concentrations of LBP in drain fluid were associated to a higher chance of AL. However, future studies are needed to validate this biomarkers [57].

Despite these limitations our study provides support to use serial measurements of CRP after elective colorectal surgery for early identification of patients at risk of developing infections, that is to say even before infection diagnosis, having CRP a lower cost of CRP compared to the PCT.

## Conclusions

Our study suggested that serial CRP measurements after elective colorectal surgery could be used as a diagnostic biomarker in the early prediction of postoperative infectious complications. A prolonged elevation of CRP levels with no subsequent decrease precedes the detection of infection by one day on average. In the present study serial PCT measurements after elective colorectal surgery were unhelpful in prediction of postoperative infectious complications.

## Authors’ original submitted files for images

Below are the links to the authors’ original submitted files for images. Authors’ original file for figure 1
