# Supplementary figures and images for: Diagnostic accuracy of C-reactive protein and procalcitonin in the early detection of infection after elective colorectal surgery – a pilot study
Source: BMC Infect Dis. 2014 Aug 16;14:444. doi: 10.1186/1471-2334-14-444 (PMC4143543; doi:10.1186/1471-2334-14-444)

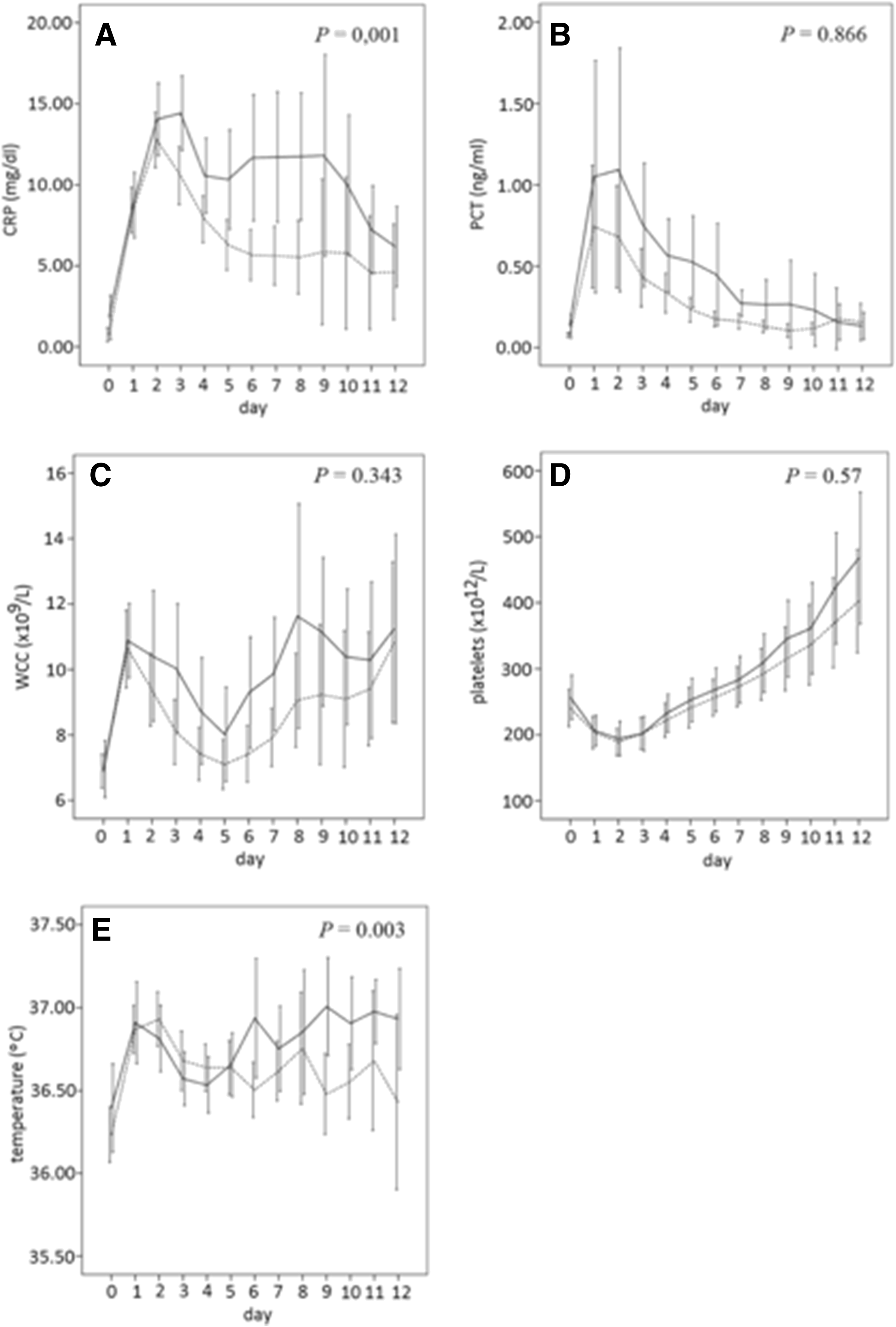

Supplement: Supplementary file 1 — Authors’ original file for figure 1 [file 12879_2014_3742_MOESM1_ESM.tiff]
